# Trends in the Use of Promotional Language (Hype) in National Institutes of Health Funding Opportunity Announcements, 1992-2020

**DOI:** 10.1001/jamanetworkopen.2022.43221

**Published:** 2022-11-21

**Authors:** Neil Millar, Bojan Batalo, Brian Budgell

**Affiliations:** 1 Department of Computer Science, University of Tsukuba, Tsukuba, Ibaraki, Japan; 2 Division of Life Sciences, Canadian Memorial Chiropractic College, Toronto, Ontario, Canada

## Abstract

This cross-sectional study examines changes from 1992 to 2020 in the use of promotional language in National Institutes of Health (NIH) funding opportunity announcements in comparison with trends reported in NIH grant applications.

## Introduction

An article in *JAMA Network Open*^1^ found that in abstracts of successful National Institutes of Health (NIH) grant applications the use of promotional language (hype) increased substantially from 1985 to 2020. As part of the discussion stimulated by the article, the question was raised as to what was motivating this change in language. In particular, some commentators questioned whether some of the responsibility for the proliferation of hype might not, in part, reside with the NIH itself.^1,2,3^ This study therefore assesses the use of hype in NIH funding opportunity announcements (FOA) from 1992 to 2020 and compares trends with those reported in grant applications.

## Methods

This study was designed to comply with relevant items of the (STROBE) reporting guideline for cross-sectional studies. Because it did not involve human participants, approval was waived by the University of Tsukuba institutional review board. A text corpus of all FOAs within the NIH archive^4^ was compiled, loaded into the CQPweb corpus analysis system,^5^ and searched for the same 139 hype adjectives previously identified in successful grant applications.^1^ For each available year (1992-2020), 2 quantitative measures were calculated: (1) the normalized frequency of the hype term (words per million [wpm]); and (2) the percentage of FOAs containing the term (dispersion). To compare longitudinal trends in FOAs with those found in grant applications, superimposed plots of the 2 time series were inspected and cross-correlation analysis was conducted using dispersion of terms in FOAs and normalized frequency in grant applications with a lag of 0 years. Correlations with a coefficient greater than 0.5 were reported with statistical significance set at *P* < .001 using a 2-tailed test. Statistical analyses were performed using R version 4.1 (R Project for Statistical Computing).^6^

## Results

A total of 16 495 FOAs were analyzed (160 151 827 words). All but 1 of the 139 hype adjectives occurred at least once in the FOAs (total occurrences 1 531 748) with the prevalence of all hype adjectives increasing by 41% from 7737 wpm in 1992 to 10 914 wpm in 2020. Adjectives showing the largest absolute increase in frequency were *key* (+1003 wpm, +327%), *senior* (+555 wpm, +1485%), *scientific* (+483 wpm, +22%), *successful* (+396 wpm, +326%) and *unique* (+276 wpm, +370%). Adjectives showing the largest increase in dispersion (ie, percentage of FOAs containing the term) were *accessible* (+70%), *meaningful* (+69%), *robust* (+67%), *accurate* (+64%) and *qualified* (+63%). A total of 37 adjectives were absent in 1992, of which the most frequent in 2020 were *sustainable* (+22 wpm), *scalable* (+19 wpm), *transformative* (+16 wpm), *transdisciplinary* (+9 wpm) and *user-friendly* (+6 wpm). Statistically significant cross-correlations between use in FOAs and grant applications were observed for 55 of the 138 hype adjectives (*r* > 0.5; *P* < .001) (Table). The Figure shows plots for the 9 most strongly correlated adjectives (*r* > 0.9) (*impactful*, *timely*, *significant*, *sustainable*, *critical*, *scalable*, *transformative*, *strategic*, *successful*).

**Table.** Hype Adjectives With Statistically Significant Cross-Correlations

Correlation coefficient	Hype adjectives	*P* value
*r* > 0.9	impactful, timely, significant, sustainable, critical, scalable, transformative, strategic, successful	All *P* < .001
0.8 < *r* <0.9	seamless, key, unmet, essential, actionable, diverse, robust, foundational, advanced, meaningful, novel, senior, strong, deeper, broad, major^a^	All *P* < .001
0.7 < *r* <0.8	transdisciplinary, urgent, powerful, ready, talented, longstanding, rigorous, tangible, accurate, groundbreaking, reproducible, intellectual, compelling^a^	All *P* < .001
0.6 <*r* <07	durable, unprecedented, accessible, unique, efficient, innovative, tremendous, quality, synergistic, efficacious, ambitious, top, extensive, cohesive, imperative^a^	All *P* < .001
0.5 < *r* <0.6	substantial, creative	All *P* < .001

^a^ Negatively correlated.

**Figure.**
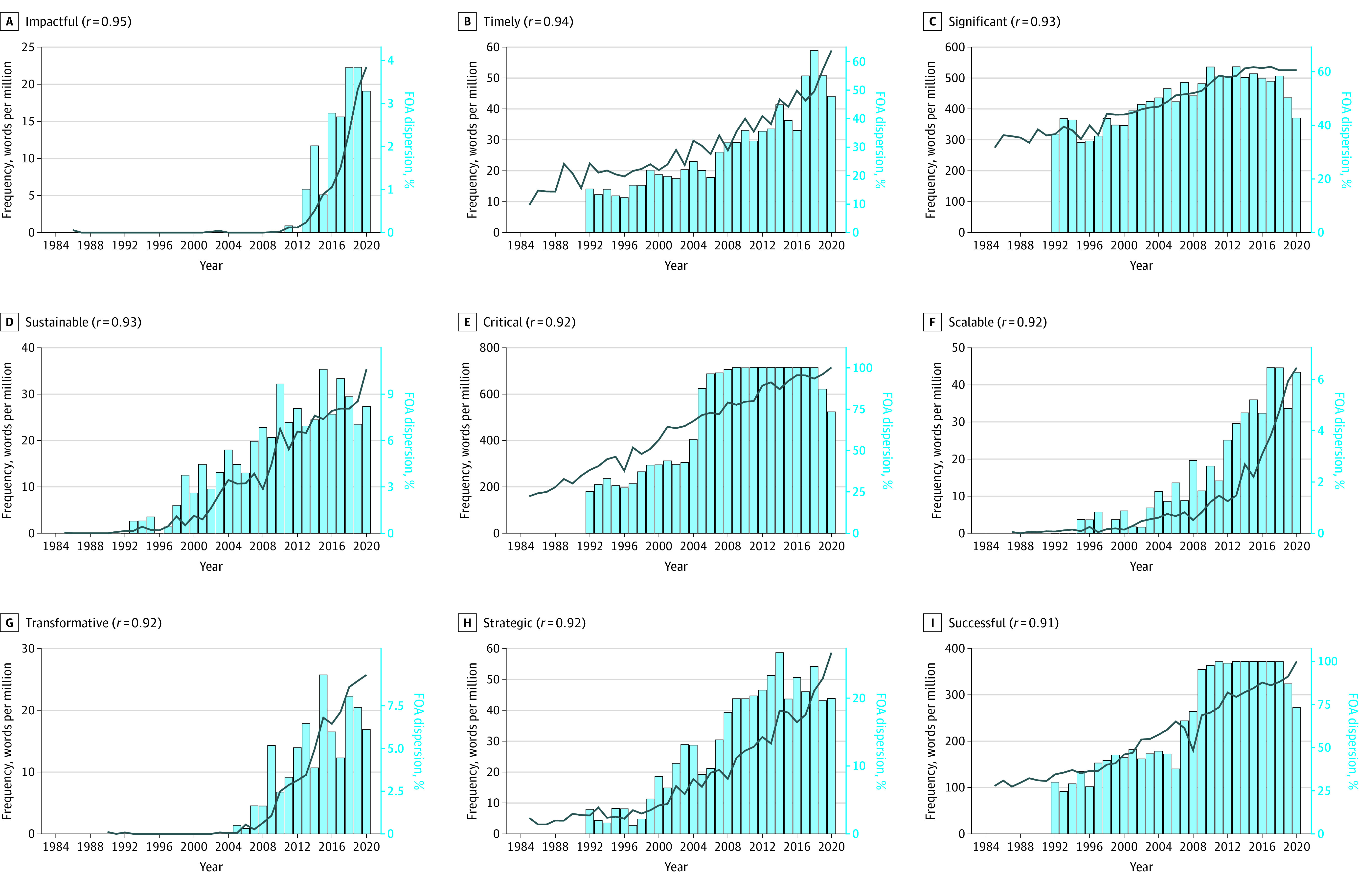
Yearly Frequency in National Institutes of Health Grant Applications and Dispersion in Funding Opportunity Announcement (FOAs) of Adjectives With a Cross-Correlation Coefficient Greater Than 0.9 Frequency of each word's use in grant applications and dispersion in FOAs is shown with the correlation coefficient between the 2 measures given in parentheses in the panel label.

## Discussion

Previous research shows that from 1985 to 2020, the use of hype terms in NIH grant applications increased substantially.^1^ This study shows that almost all the same terms (138 out of 139) are also used by the NIH in funding opportunity announcements. In FOAs, from 1992 to 2020, the overall use of these hype terms increased, and, for many individual terms (55 out of 138), the patterns of change are correlated with those in grant applications. Among the strongest correlations are those adjectives that increased rapidly and suddenly in popularity (eg, *impactful*, *scalable*, *sustainable*, *transformative* ), and have been described elsewhere as buzzwords.^1^ These results suggest that increase in the use of hype language in grant applications may, in part, be a response to instructions from the NIH. One limitation is that in the context of FOAs some adjectives might not represent hype (eg, *scalable, successful, key*). Furthermore, the analysis compared overall trends without assessing the relationship between individual FOAs and corresponding applications.

## Notes

Supplement.
Data Sharing Statement
