# Supplementary material for: Trends in the Use of Promotional Language (Hype) in National Institutes of Health Funding Opportunity Announcements, 1992-2020
Source: JAMA Netw Open. 2022 Nov 21;5(11):e2243221. doi: 10.1001/jamanetworkopen.2022.43221 (PMC9679874; doi:10.1001/jamanetworkopen.2022.43221)
Supplement: Supplement. — Data Sharing Statement [file jamanetwopen-e2243221-s001.pdf]

Data Sharing Statement

Millar. Trends in the Use of Promotional Language (Hype) in National Institutes of Health Funding Opportunity Announcements, 1992-2020. *JAMA Netw Open*. Published November 21, 2022. doi:10.1001/jamanetworkopen.2022.43221

Data
Data available: Yes
Data types: Data (not involving human participants)
How to access data: millar.neil.gm@u.tsukuba.ac.jp
When available: With publication

Supporting Documents
Document types: None

Additional Information
Who can access the data: anyone requesting the data
Types of analyses: for replication of these findings
Mechanisms of data availability: without investigator support
